# Comparison of Six Commercial Meat Starter Cultures for the Fermentation of Yellow Mealworm (*Tenebrio molitor*) Paste

**DOI:** 10.3390/microorganisms7110540

**Published:** 2019-11-08

**Authors:** Borremans An, Crauwels Sam, Vandeweyer Dries, Smets Ruben, Verreth Christel, Van Der Borght Mik, Lievens Bart, Van Campenhout Leen

**Affiliations:** 1 Lab4Food, Department of Microbial and Molecular Systems (M2S), KU Leuven, Campus Geel, B-2440 Geel, Belgium; an.borremans@kuleuven.be (B.A.); dries.vandeweyer@kuleuven.be (V.D.); ruben.smets@kuleuven.be (S.R.); mik.vanderborght@kuleuven.be (V.D.B.M.); 2 Laboratory for Process Microbial Ecology and Bioinspirational Management (PME&BIM), Department of Microbial and Molecular Systems (M2S), KU Leuven, Campus De Nayer, B-2860 Sint-Katelijne-Waver, Belgium; sam.crauwels@kuleuven.be (C.S.); christel.verreth@kuleuven.be (V.C.); bart.lievens@kuleuven.be (L.B.)

**Keywords:** fermentation, mealworm paste, bacterial community dynamics, microbial counts, quantitative PCR, metabolite target analysis

## Abstract

In this study, six commercial meat starters, each consisting of a pure strain of a lactic acid-fermenting bacterium (including *Lactococcus lactis*, *Lactobacillus curvatus*, *L. farciminis*, *L. plantarum*, *L. sakei*, and *Pediococcus acidilactici*), were tested for their ability to ferment a paste produced from the yellow mealworm (*Tenebrio molitor*). During fermentation, microbial counts, pH, and the bacterial community composition were determined. In addition, UPLC-MS was applied to monitor the consumption of glucose and the production of glutamic (Glu) and aspartic (Asp) acid. All tested starters were able to ferment the mealworm paste, judged by a pH reduction from 6.68 to 4.60–4.95 within 72 h. Illumina amplicon sequencing showed that all starters were able to colonize the substrate efficiently. Moreover, the introduction of the starter cultures led to the disappearance of *Bacillus* and *Clostridium* species, which were the dominant microorganisms in un-inoculated samples. Of the six cultures tested, *Lactobacillus farciminis* was most promising as its application resulted in the largest increase (±25 mg/100 g of paste) in the content of free glutamic and aspartic acid. These amino acids are responsible for the appreciated umami flavour in fermented food products and might stimulate the acceptance of insects and their consumption.

## 1. Introduction

Interest exists in the introduction of insects in the Western food pattern, but a number of hurdles prevent the large scale use of insects in foods. With respect to legislation, insects are considered a novel food according to Reg. (EU) No. 2015/2283 and therefore authorisation is required. Currently, a number of dossiers are under evaluation. In addition to legislation, insects and insect-based matrices show the problem that their shelf life is limited. For instance, De Smet et al. [1] observed pronounced microbial growth in mealworm paste during three weeks of storage at 4 °C, even when preservatives were included. In addition, the taste of insects is not very distinct and does not (yet) convince a large number of consumers to regularly purchase and eat insects or insect-based foods [2,3,4,5]. In the same way as for traditional meat sources, fermentation may present a technology to improve both shelf life and taste of insects. It is possible that shelf life can be improved if the pH can be reduced sufficiently and fast enough by fermentation to prevent spoilage organisms from growing, and that taste can be intensified by the generation of specific amino acids yielding a desired taste, such as glutamic acid (Glu) and aspartic acid (Asp) responsible for the umami taste [6,7]. Introducing the umami taste in insect-derived food products or edible food components might stimulate their acceptance and consumption, because this taste is well appreciated in the meat consuming Western culture.

Klunder et al. [8] described the fermentation of mixtures of sorghum containing 10% and 20% of roasted mealworms (% d.m. of composite meal) with a natural starter obtained by backslopping of the meal. Proof-of-concept for the fermentation of a 100% mealworm-based matrix, i.e., finely fragmented mealworms, using a commercial meat starter Bactoferm^TM^ F-LC (Chr. Hansen, mixture of *Pediococcus acidilactici*, *Lactobacillus curvatus,* and *Staphylococcus xylosus*) was given by Borremans et al. [9] and De Smet et al. [1]. It was shown to rapidly reduce the pH of the mealworm paste. It is not known, however, whether other meat starter cultures are also suitable to ferment mealworm paste, and how they perform with respect to pH reduction, impact on the background microbial dynamics and Glu and Asp generation. Therefore, the aim of this work was to test the fermentation of finely fragmented mealworms with other meat starter cultures.

In contrast to the previously tested mixed culture, the starters tested in this work all contain one single bacterial strain belonging to the lactic acid bacteria (LAB), a microbial subgroup present in most meat starters [10,11]. LAB play a key role in numerous industrial and artisanal food fermentations. Their fermentative metabolism prevents the development of spoilage and pathogenic organisms by acidification of the product, but LAB also contribute to product characteristics such as flavour and texture [12]. In addition, certain LAB have been found to produce antimicrobial compounds, such as bacteriocins, and they are used as a bioprotective agent to preserve fresh and processed meat and fish [13,14]. Some meat starters, such as the starter used in the previous work, contain Gram-positive, catalase-positive cocci that contribute to the sensory quality of fermented sausages. They participate in desirable reactions during ripening, such as decomposition of peroxides, colour stabilisation, proteolysis, and lipolysis [10].

In meat fermentations, the addition of the curing agent nitrite is important as it aids in colour development, it inhibits the growth of *Salmonella* and *Clostridium*, it prevents lipid rancidity, and it produces the typical cured flavour [15]. The questions can be raised whether the addition of nitrite is necessary in mealworm fermentations and what the effect is on the performance of the meat starter cultures. Therefore, for the control without starter and for all starters considered, fermentation was performed both on pastes without and with nitrite.

## 2. Materials and Methods

### 2.1. Sample Materials

*Tenebrio molitor* larvae, originating from two production batches, were purchased from Nusect (Nusect BV, Sint-Eloois Winkel, Belgium), a company producing insects for food and feed. The larvae were killed by freezing and stored at −18 °C until further use. The starter cultures used in this study were kindly provided by Chr. Hansen (Chr. Hansen A/S, Hoersholm, Denmark), who selected six pure LAB cultures (*Lactobacillus plantarum, L. farciminis, L. sakei, Pediococcus acidilactici, L. curvatus,* and *Lactococcus lactis*) that match best for their nutrient requirements with the chemical composition of the larvae (i.e., contents of macronutrients, micronutrients, vitamins, and minerals as described by Nowak et al. [16]). The starters, acquired in a ready-to-use and highly concentrated form, were stored at −18 °C.

### 2.2. Experimental Design, Fermentation Procedure, and Online pH Measurement

Seven series of fermentations were carried out with larvae originating from the first production batch, involving one series that was not inoculated (negative control) and six series that were inoculated with one of the aforementioned starter cultures. Afterwards, in order to investigate the repeatability of the fermentation when using other batches of mealworms, the fermentations with *P. acidilactici* and *L. sakei* were performed with a second batch of mealworms. 

Mealworm paste was produced and fermented as described before [9] with a slight modification. Briefly, frozen larvae were blanched (40 s), mixed into a paste using a kitchen mixer (Bosch CNHR 25, 2 min), and inoculated with one of the cultures (except for the negative control). The freeze-dried cultures were added to reach a level of ±6.5 log cfu/g, similar to meat fermentations [17]. After the addition of NaCl (2.8%, *w*/*w*) and d(+)-Glucose (0.75%, *w*/*w*), a series of 50 mL Falcon tubes was completely stuffed with 50 g paste and closed tightly with the lid to obtain (semi-) anaerobic conditions, and then placed at 35 °C for seven days. For the experiments involving nitrite, pastes were supplemented with 0.015% NaNO_2_ (*w*/*w*) prior to stuffing the Falcon tubes. At three time points, i.e., at zero (after inoculation), three, and seven days of fermentation, three Falcon tubes were withdrawn for microbiological analysis. The remainder of the samples was frozen at −18 °C for metabolite target analysis, 16S rRNA gene amplicon sequencing, and qPCR.

In addition to the three Falcon tubes necessary for each sampling point, one additional tube of each condition was prepared. Online pH monitoring was performed by inserting a pH electrode (pHenomenal pH 1100H with SenTix 82 pH electrode, VWR, Leuven, Belgium) through a puncture in the lid of that tube. The hole had a diameter that corresponded exactly to the diameter of the electrode and parafilm tape was applied at the entrance of the electrode in the lid. The electrode was inserted 10 cm in the paste and pH was recorded throughout the fermentation.

### 2.3. Microbiological Plate Counts

Microbiological plate counts were performed according to the ISO standards for microbial analyses of food as described by Dijk et al. [18]. To this end, 5 g of fermented mealworm paste were added to 45 mL of sterile peptone physiological salt solution (0.85% (*w*/*v*) NaCl, 0.1% (*w*/*v*) peptone, Biokar Diagnostics, Beauvais, France) and homogenized in a stomacher bag (Bagmixer^®^ 400 W, Interscience, Saint Nom, France) for 1 min. Ten-fold dilutions were plated on Plate Count Agar (PCA, Biokar Diagnostics, incubation at 30 °C for 72 h) for the determination of the total viable aerobic and anaerobic count, on de Man Rogosa Sharpe agar (MRS, Biokar Diagnostics, incubation at 30 °C for 72 h) for presumptive LAB, and on Iron Sulphate Agar (ISA, Thermo Fisher Scientific, Merelbeke, Belgium, incubation at 37 °C for 48 h) for presumptive sulphite reducing clostridia (SRC). Anaerobic conditions were created using anaerobic jars (VWR), gas generation kits, and indicator strips (Thermo Fisher Scientific). Aerobic bacterial endospores were determined by giving the 10^−1^ dilution a heat-shock treatment (10 min at 80 °C), followed by plating a ten-fold serial dilution on PCA. 

### 2.4. Bacterial Community Analysis Yielding Relative Abundances

The bacterial community composition of the samples were determined using Illumina MiSeq sequencing of partial 16S rRNA gene amplicons. To this end, the microbial genomic DNA of each sample was extracted following the protocol of the PowerSoil DNA isolation kit (MO BIO Laboratories, Carlsbad, CA, USA). DNA quality and concentrations were assessed using a Nanodrop Spectrophotometer (mySPEC, VWR, Leuven, Belgium), and all extracted DNA was stored at −18 °C for further amplifications. Sequencing of the 16S rRNA amplicons was performed using the dual index strategy as described by Kozich et al. [19]. Briefly, DNA samples were subjected to a PCR assay using barcode-labelled versions of the primers 515F (5′-GTGCCAGCMGCCGCGGTAA-3′) and 805R (5′-GGACTACHVGGGTWTCTAAT-3′) [20], amplifying the V4 region (250 bp) of the 16S rRNA gene. The PCR amplification (20 µL) contained 1 µL of 1:10 diluted DNA, 1.5 µL of dNTP mix (0.15 mM each), 0.5 µL of each primer (0.5 µM), 2 µL of Titanium Taq PCR buffer, 0.4 µL of Taq DNA polymerase (Clontech, Saint-Germain-en-Laye, France), and 14.1 µL of sterile milli-Q water. PCR amplification was performed with a Thermal Cycler (T100^TM^, Bio-Rad, Temse, Belgium) using the following program: One cycle at 95 °C for 2 min, 30 cycles of denaturation at 95 °C for 45 s, primer annealing at 59 °C for 45 s, and a final extension step at 72 °C for 10 min. Following amplification, the PCR products were purified by Agencourt AMPure XP beads (Beckman Coulter, Brea, CA, USA) and quantified using the Qubit fluorometer (HS reaction kit, Invitrogen, Carlsbad, CA, USA). The quantified PCR products were combined into a library in equimolar concentrations and subjected to an ethanol precipitation. As a final quality control, the pooled library was subjected to electrophoresis on an agarose gel (1.5%), after which amplicons were cut out and purified with the NucleoSpin gel and PCR cleanup kit (Machery-Nagel, Düren, Germany). Finally, the library was diluted to 2 nM and sequenced at the Center of Medical Genetics Antwerp (University of Antwerp, Antwerp, Belgium) using an Illumina MiSeq sequencer (V2 500 cycle kit, Illumina, San Diego, CA, USA). 

Resulting sequences were received as a demultiplexed FASTQ file (data deposited in a Sequence Read Archive; BioProject accession PRJNA576139). Paired-end reads were merged using USEARCH (v10.0.240; [21]) to form consensus sequences originating from each sample. A maximum number of ten mismatches was allowed in the overlap region. Next, sequences were truncated at the 250th base and shorter reads or reads with an expected error threshold above 0.02 were discarded. In order to remove potential contaminant sequences, sequences were classified using the “classify.seqs” command in Mothur (v.1.39.3; [22]) against the Silva taxonomy database (release v1.2.3; [23]). Sequences originating from chloroplasts or mitochondria or sequences identified as “eukaryote” or “unknown” were deleted with Mothurs “remove.lineage” command. With the UNOISE algorithm [23] command implemented in USEARCH [24], further error correction (denoising) was performed as follows: (i) Reads with sequencing errors were identified and corrected, (ii) chimeras were removed, and (iii) PhiX were removed. Due to uneven sequencing depth across samples, the number of sequences was rarefied to 11,561 sequences per sample. A sequence table containing all amplicon sequence variants (ASV) was constructed and the taxonomic origin of each ASV was determined with the SINTAX algorithm in USEARCH [25], on the Silva Living Tree Project v123 (LTP v123) database. Additionally, ASV representative sequences (selected by UPARSE) were subjected to a BLAST search [26] against GenBank [27], excluding uncultured or environmental entries. Primarily, assignments were based on SINTAX results, but BLAST results were used when SINTAX assignment was inconclusive. Diversity indices calculations were performed on the microbial communities of inoculated and un-inoculated samples using Uparse (v10.0.240, R-package, R Development Core Team). Relative ASV abundances and diversity indices were averaged over three samples per fermentation. ASVs present in a relative abundance of at least 1% and belonging to the same genus or phyla were combined and presented in the same colour. However, an exception was made for the ASVs belonging to the added starters.

### 2.5. Quantitative Real-Time PCR Amplification to Determine Absolute Abundances 

Illumina MiSeq sequencing yielded relative abundances. In order to obtain absolute abundances, quantitative real-time PCR (qPCR) was used to assess the total bacterial DNA in the samples. The gene copy number of each sample was then used to convert the relative abundances into absolute abundances. Amplification and detection of the total target DNA by real-time PCR were performed with a QuantStudio3 real-time PCR system (Applied Biosystems, Thermo Fisher Scientific, Asse, Belgium) using the primers 515F and 805R. Each PCR reaction was carried out in volumes of 10 µL containing 1 µL of 1:10 diluted DNA, 0.25 µL of each primer (0.2 µM each), 5 µL of PowerUp^TM^ SYBR^®^ Green qPCR master mix (Life technologies, Thermo Fisher Scientific, Asse, Belgium) and 3.5 µL sterile milli-Q water. The qPCR reactions were performed under thermal cycler conditions of 2 min at 50 °C and 10 min at 95 °C, followed by 40 amplification cycles of 15 s at 95 °C and 1 min at 60 °C, with collection of fluorescence signal at the end of each cycle. Following amplification, melting temperature analysis was performed to determine the specificity of the PCR. In each analysis, a positive control, a negative control (DNA replaced by sterile milli-Q water), and a standard (serial dilution of cleaned PCR products from paste inoculated with the starter *P. acidilactici*) was included. All template DNA of the fermentations was tested in triplicate on each plate, whereas the standard run was performed in duplicate. The gene copy number of each sample was calculated with the Connect^TM^ Software (Thermo Fisher Scientific) and expressed as gene copies/µL DNA extract.

### 2.6. Metabolite Target Analysis

Ultra-performance liquid chromatography–mass spectrometry (UPLC-MS) was applied for determination of the concentrations of glucose (to monitor the consumption of glucose) and the free amino acids (FAA) Asp and Glu in the samples. The remainder of the three samples used for the microbiological analyses was pooled together to obtain enough material for the sample preparation procedure and subsequent UPLC-MS analysis. The sample preparation procedure consisted of a freeze drying step (Lyovapor^TM^ L-200, Büchi, Flawil, Switzerland) and a subsequent Soxhlet fat extraction with petroleum ether (VWR). Following the defatting, 0.100 g of each defatted sample was weighed to the nearest 0.0001 g into 15 mL centrifuge tubes, and 10.0 mL 0.1 N HCl (Puris. P.a, ACS Reagent grade, Sigma-Aldrich, Overijse, Belgium) was added to solubilise the FAA and glucose. The resulting mixtures were heated to 40 °C, sonicated for 5 min at 40 °C, and finally centrifuged at 4000× *g* for 10 min. To precipitate the proteins, an aliquot of 500 µL supernatant was taken from the samples after centrifugation and thoroughly mixed with 500 µL of 10% (*w*/*v*) sulfosalicylic acid (SSA, Acros Organics, Thermo Fisher Scientific, Geel, Belgium). The samples were chilled on ice and centrifuged (17,000 × *g* at 4 °C for 10 min). For amino acid analysis, free Asp and Glu were derivatized by adding a mixture of 70 µL of a 0.08 M (pH = 8.8) borate buffer (Acros Organics) and 20 µL of 6-aminoquinoyl-N-hydroxysuccimidyl carbamate (AQC) reagent to 10 µL of the obtained clear supernatant of the centrifuged samples. The samples were mixed and incubated for 10 min at 55 °C. For glucose analysis, an aliquot of 50 µL of the supernatant was taken and thoroughly mixed with 950 µL LC-MS grade acetonitrile (Biosolve, Valkenswaard, the Netherlands). 

Sample analysis was performed on a Waters Acquity UPLC H-Class system (Waters Corp., Milford, MA, USA) equipped with a quaternary solvent manager, an autosampler maintained at 4 °C, and a Waters QDA mass detector operating in selected ion recording (SIR) mode. The ESI source was used in positive (FAA) or negative (glucose) mode with the following conditions: The ion source capillary voltage was kept at 0.8 kV, the cone voltages were 10 and 4 V for FAA and glucose analysis, respectively, and the desolvation temperature was 600 °C. The separation of FAA was performed on a Waters Cortecs^®^ UPLC C18 column (2.1 mm × 150 mm, 1.6 µm) which was maintained at 55.0 ± 0.5 °C during the runtime of 12 min. The amino acids were eluted using a nonlinear gradient of ultrapure water (A) and acetonitrile (B), both containing 0.1% formic acid as modifier. Formic acid and acetonitrile were obtained from Biosolve. Glucose was separated isocratically on a Waters Acquity UPLC BEH amide column (2.1 mm x 150 mm, 1.7 µm particles) with a 10/90 mixture of ultrapure water/acetonitrile containing 0.1% NH_4_OH (HiPerSolv CHROMANORM, VWR, Leuven, Belgium) as modifier. The column temperature was maintained at 70.0 ± 0.5 °C during the runtime of 3 min. Before sample injection (injection volume of 1 µL), the system was calibrated using analytical standards bought from Sigma-Aldrich (FAA) and Thermo Fisher Scientific (glucose). Samples were analysed in duplicate and the results are represented as the average of these two measurements. The Empower^TM^ 3 software (Waters Corp., Milford, MA, USA) was used to obtain calibration curves and subsequently quantify the FAA Asp and Glu and glucose in the defatted samples.

### 2.7. Statistical Analyses

Statistical analyses were conducted using SPSS Statistics (IBM SPSS Statistics version 25, New York, NY, USA). One-way ANOVA was performed to test variation of the microbial counts, pH measurements, alpha diversity indices (ASV richness and the Shannon–Wiener diversity index), and bacterial 16S rRNA gene copies of the total bacteria among the different starters and the control experiment. When necessary, pairwise comparison was performed using Tukey’s post-hoc test. All tests considered a 0.05 significance level.

## 3. Results and Discussion

### 3.1. Acidification

**Figure 1:**
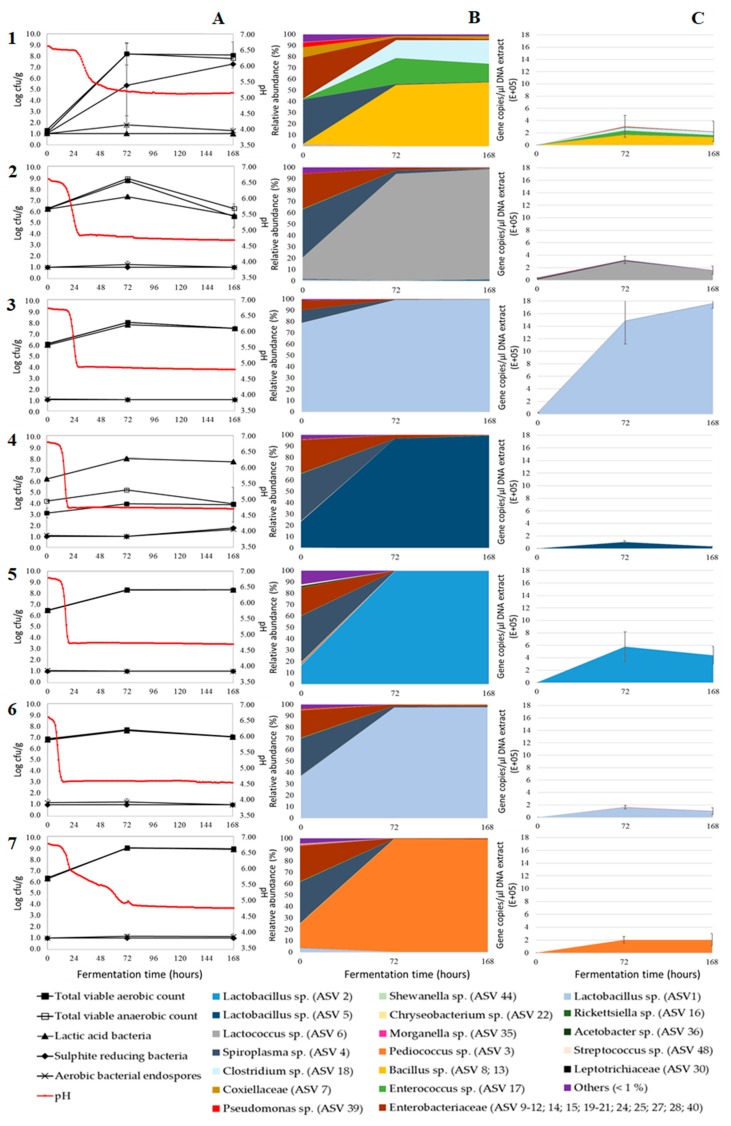
Microbial counts and pH (**A**), relative abundance in the bacterial community (**B**), and estimated absolute abundances (EAA) (**C**) during fermentation of pastes without sodium nitrite prepared from mealworms of the first production batch, and without starter (control experiment) (**1**) or inoculated with starter *Lactococcus lactis* (**2**), *Lactobacillus curvatus* (**3**), *L. farciminis* (**4**), *L. plantarum* (**5**), *L. sakei* (**6**), or *Pediococcus acidilactici* (**7**). The microbial counts and values of relative abundance and EAA are the mean of analyses performed on three replicate samples. Error bars represent the standard deviation. Only amplicon sequence variants (ASVs) represented by an average relative abundance of more than 1% of the sequences in any sample are shown. Other ASVs are grouped together in “others (<1%)”.

The evolution of the pH during the seven-day incubation period of the pastes is shown in Figure 1A. All pH profiles show a clear acidification in function of time, both in the negative control and when a starter culture was added. The initial pH of the matrix was approximately 6.68. For the negative control sample, this was maintained for about 33 h before the pH decreased during two days to an end value of 5.17. For the inoculated matrices, acidification started after eight to 22 h. The subsequent pH drop resulted in a pH ranging from 4.60 to 4.95 after three days. Upon further fermentation, the pH remained constant for all fermentations. 

**Figure 2:**
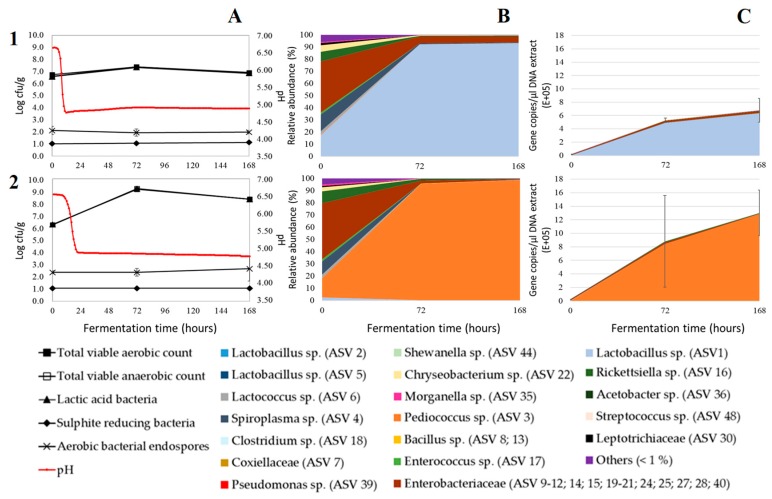
Microbial counts and pH (**A**), relative abundance in the bacterial community (**B**), and estimated absolute abundances (EAA) (**C**) during fermentation of pastes without sodium nitrite prepared from mealworms of the second production batch, and inoculated with starter *Lactobacillus sakei* (**1**) or *Pediococcus acidilactici* (**2**). The microbial counts and values of relative abundance and EAA are the mean of analyses performed on three replicate samples. Error bars represent the standard deviation. Only amplicon sequence variants (ASVs) represented by an average relative abundance of more than 1% of the sequences in any sample are shown. Other ASVs are grouped together in “others (<1%)”.

The ability of starters to cause a fast acidification is an important characteristic, since it has an impact on the safety, aroma, and flavour of the fermented product [28]. For mealworms, which are susceptible to microbial spoilage due to their high concentration of nutrients and high water activity (±0.96 according to Vandeweyer et al. [29]), a rapid acidification is essential to prevent the growth of pathogens and spoilage organisms in the first stage of the fermentation. As shown in Figure 1A, there were differences in the acidification by the various tested starters. All inoculated samples, except the samples inoculated with *P. acidilactici*, showed a rapid acidification. *L. sakei* acidified the paste the fastest, followed by *L. farciminis*, *L. plantarum*, *L. lactis,* and *L. curvatus*. Fermentation with *P. acidilactici* resulted in a slow, irregular acidification. However, when this fermentation was repeated using larvae from a different production batch, a rapid acidification was obtained as well, as shown in Figure 2A. 

**Figure A1:**
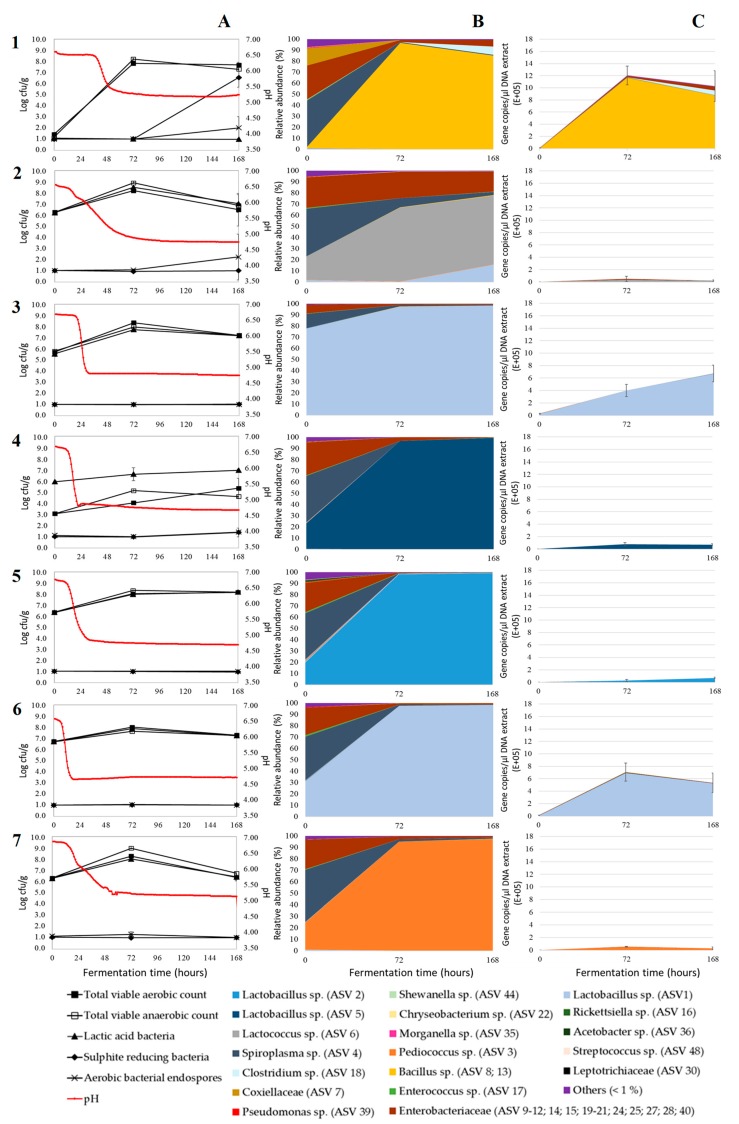
Microbial counts and pH (**A**), relative abundance in the bacterial community (**B**), and estimated absolute abundances (EAA) (**C**) during fermentation of pastes with addition of sodium nitrite and prepared from mealworms of the first production batch, and without starter (control experiment) (**1**) or inoculated with starter *Lactococcus lactis* (**2**), *Lactobacillus curvatus* (**3**), *L. farciminis* (**4**), *L. plantarum* (**5**), *L. sakei* (**6**), or *Pediococcus acidilactici* (**7**). The microbial counts and values of relative abundance and EAA are the mean of analyses performed on three replicate samples. Error bars represent the standard deviation. Only amplicon sequence variants (ASVs) represented by an average relative abundance of more than 1% of the sequences in any sample are shown. Other ASVs are grouped together in “others (<1%)”.

**Figure A2:**
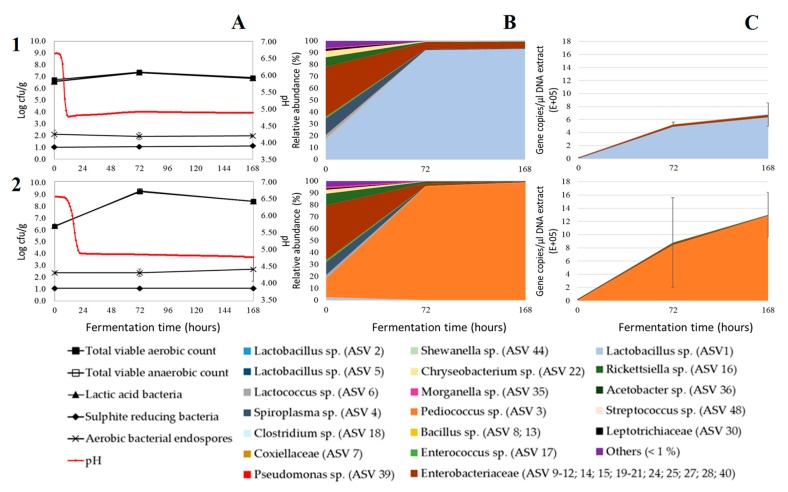
Microbial counts and pH (**A**), relative abundance in the bacterial community (**B**), and estimated absolute abundances (EAA) (**C**) during fermentation of pastes with addition of sodium nitrite and prepared from mealworms of the second production batch, and inoculated with starter *Lactobacillus sakei* (**1**) or *Pediococcus acidilactici* (**2**). The microbial counts and values of relative abundance and EAA are the mean of analyses performed on three replicate samples. Error bars represent the standard deviation. Only amplicon sequence variants (ASVs) represented by an average relative abundance of more than 1% of the sequences in any sample are shown. Other ASVs are grouped together in “others (<1%).

Similar pH profiles were obtained when sodium nitrite was added to the paste (Figure A1-A and Figure A2-A, Supporting Information). The reduction in pH, however, was generally initiated later compared to the corresponding samples from the fermentation with the same starter but without sodium nitrite. Korkeala [30], who investigated the effect of sodium nitrite on the growth of LAB, observed an increasing lag phase when the amount of added sodium nitrite was gradually increased from 0 to 400 mg/L. This increasing lag phase can explain the delay in acidification, as acid production mainly occurs during the exponential phase of the starter [31]. Despite the different acidification rates of the starters and the delay in acidification when nitrite was used, all fermentations with inoculation reached pH values below 5.10 at day three of the fermentation, which is considered to be an important threshold in the meat industry from a food safety perspective [17].

### 3.2. Microbial Plate Counts

The microbiological counts of the fermentations with inoculation differed largely from those of the negative control experiment (Figure 1A). At the start of the negative control experiment, all counts were low (below 1.5 log cfu/g). Even though blanching, here applied as a pretreatment before fermentation, generally involves a large reduction in the microbial load of edible insects [32], the process was not enough to prevent the outgrowth of undesired SRC in the control. The SRC count increased exponentially during fermentation, resulting in counts of up to 7.5 log cfu/g at the end of the fermentation. This high SRC count was accompanied by an unpleasant smell of the larvae paste. The addition of sodium nitrite to the paste delayed, but did not prevent this problem (Figure A1-A, Supporting Information). From the third day and onwards of the control experiment, the counts on PCA were up to 9.5 log cfu/g for the aerobic as well as the anaerobic organisms, and 1.5 log cfu/g for bacterial endospores. Selective plating of the control on MRS yielded values below the detection limit which indicates the absence of presumptive LAB. This is in contrast with earlier research [9], where growth of LAB was observed in un-inoculated paste in two out of three fermentations.

The microbiological counts of the fermentations with inoculation were all very similar to each other. As can be expected, LAB counts of all fermentations with inoculation were high. At the start of the fermentations, LAB counts ranged between 5.9 and 6.9 log cfu/g, corresponding with the added amount of starter. The presumptive LAB grew substantially (*p* < 0.05) during the first three days of the fermentation, resulting in LAB counts of up to 9.1 log cfu/g. Subsequently, the LAB counts remained constant throughout the entire fermentation or decreased to a minimum of 5.0 log cfu/g (Figure 1A). The growth of presumptive LAB can be influenced by the presence of nitrite, as shown in Figure A1-A (Supporting Information). For a third of the starters, substantially (i.e., differences of more than 1.0 log cfu/g) lower LAB counts were observed at day three compared to the corresponding samples from the fermentation with the same starter but without sodium nitrite. If present in large amounts, nitrite can inhibit the growth of some lactic acid bacteria. However, with the amounts regularly used in meat industry, it is usually not the case [33,34]. In addition, if inhibition takes place, nitrite seems to affect the lag phase rather than the exponential growth phase. The PCA counts (total aerobic and anaerobic counts) during fermentation were very similar to the MRS counts, indicating the predominance of LAB. Only for the starter *L. farciminis* low total counts were observed compared to the LAB counts, which could not be explained. Other microbial counts (aerobic bacterial endospores and sulphite reducing clostridia) were low during fermentation for all inoculated samples, mostly below the detection limit of 1.0 log cfu/g. 

Statistical analysis showed differences (*p* < 0.05) in the growth of the presumptive LAB during fermentation. After three days of fermentation, the fermentation inoculated with the starter *P. acidilactici* showed the strongest growth of presumptive LAB (±2.7 log cfu/g), while the LAB counts of the fermentation inoculated with *L. sakei* increased the least (±0.8 log cfu/g). This result was in line with Sriphochanart and Skolpap [35], who observed a better growth of *P. acidilactici* in Thai fermented sausages compared to *L. sakei*. Repeating those two fermentations with larvae from a different production cycle had little to no influence on the results (±2.9 log cfu/g and ±0.8 log cfu for *Pediococcus acidilactici* and *Lactobacillus sakei*, respectively), as shown in Figure 2A. 

The results of the microbial counts and pH measurements indicate that all starters are able to ferment the larvae paste, i.e., they all show a rapid acidification, good growth of the LAB, and inhibition of undesirable SRC. Microbial counts, however, do not give ample information on the composition of the microbiota. It is possible, for example, that LAB other than the added starter grow during fermentation. Therefore, high-throughput 16S rRNA gene sequencing was used to unravel the bacterial community dynamics during fermentation and to evaluate whether the starters can outcompete the endogenous bacteria.

### 3.3. Relative Bacterial Abundances and Bacterial Diversity

The sequencing yielded a total of 286 amplicon sequence variants (ASVs), defined as unique biological sequences obtained after a denoising step, over all samples. Table 1 shows two alpha diversity metrics, i.e., the ASV richness and the Shannon–Wiener diversity index. The ASV richness indicates the number of ASVs observed in a sample, while the Shannon–Wiener index describes diversity not only in terms of the richness but also in terms of the relative abundance of the ASV. More specifically, a higher Shannon value means more diversity [36]. All samples showed an observed ASV amount between 55 and 130 at day zero and between seven and 55 at day three and day seven of the fermentation. The Shannon–Wiener index varied between 0.91 and 2.94 at day zero and between 0.02 and 1.54 at day three and seven. Both diversity indices decreased significantly (*p* < 0.05) during fermentation, indicating that the diversity at the start of the fermentation was higher than the diversity at the end of the fermentation. This decrease in diversity was also observed in other fermented food products when a starter culture was used, such as sauerkraut [37], and it was also observed when sodium nitrite was added to the mealworm pastes (Table A1, Supporting Information). 

The dynamics of the most abundant ASVs, represented by more than 1% of the sequences in any sample, during the seven-day incubation period of the inoculated and un-inoculated mealworm paste are shown in Figure 1B. In all experiments, a bacterium related to the species *Spiroplasma* was abundantly present at the start (up to 42.2%, ASV 4). *Spiroplasma* was already found in fresh [38,39,40,41] and in processed mealworms previously [38], and it may be a bacterium typically associated with this insect species. It is typically found as an endosymbiont in the insect gut [42], and it is generally not considered as a foodborne pathogen [41]. In addition, several ASVs belonging to the family of the *Enterobacteriaceae* were found at the start of all fermentations (ASVs 9–12, 14, 15, 19–21, 24, 25, 27, 28, and 40). Detection of these ASVs in a high abundance (in total ranging from 8.5% to 45.9%) was unexpected, since the larvae were subjected to a blanching step prior to fermentation and *Enterobacteriaceae* are known to be quite sensitive to heat treatments [43]. An explanation can be that the blanching step, although reducing microbial numbers as shown by Vandeweyer et al. [32], did not destroy the DNA of dead microorganisms. On the other hand, due to the nutrient-rich matrix, the high water activity (±0.96) and the high microbial load after inoculation (±6.5 log cfu/g), it can also be assumed that the DNA of dead bacteria after the blanching step is broken down quickly.

At day three of the fermentation, a significant shift in the bacterial ecology was observed in both the control experiment as in the inoculated fermentations. The control samples harboured substantial quantities of *Bacillus* sp. (up to 57.7%, ASVs 8 and 13) and *Clostridium* sp. (up to 21.1%, ASV 18). Endospore formers such as *Bacillus* sp. and *Clostridium* sp. were previously encountered in edible insects, including mealworms [38,41,44,45]. Both genera are of concern regarding food safety, since they contain the food pathogens *C. perfringens*, *C. botulinum,* and *B. cereus* [42]. An *Enterococcus* species (up to 23.2%, ASV 17) occurred dominantly alongside with *Bacillus* and *Clostridium*. Although some species of *Enterococcus* are pathogenic, other species have been isolated from fermented foods and serve as probiotics [46]. When sodium nitrite was added to the paste, the growth of *Clostridium* sp. and *Enterococcus* sp. was suppressed, resulting in the dominance of *Bacillus* sp. in the control experiment (up to 96.5%, Figure A1, Supporting Information). Analysis of the inoculated samples suggested the dominance of the applied starter strains from day three of the fermentation and onwards, with their relative abundances ranging between 94.0% and 99.8%. Although six starter cultures were tested, only five different ASVs were recovered that were dominating the fermentations: Three *Lactobacillus* species (ASVs 1, 2, and 5), one *Pediococcus* species (ASV 3), and one *Lactococcus* species (ASV 6). More specifically, the starter cultures *L. sakei* and *L. curvatus* were assigned to the same ASV (ASV1), as shown in Figure 1B. The starter *L. sakei* has been described earlier as closely related to *L. curvatus*, *L. fuchuensis*, and *L. graminis* [47]. Moreover, the existence of this clade was confirmed through comparison of the genome sequences available for several strains belonging to these species [48,49]. Hence, the 250 bp read length of the investigated 16S rRNA gene amplicon was too short to distinguish between these two closely related species. Using an alternative marker such as a protein coding gene, preferably a house-keeping gene, could help discriminate these related species in future research [50].

The sequencing results and the microbial numbers are in line with each other, pointing at a high share of LAB in the bacterial load of the inoculated samples and a high share of *Clostridia* in the bacterial load of the control experiment. Although the bacterial community composition may differ largely between different rearing cycles or production batches, as demonstrated by Vandeweyer et al. [29], the differences in the original microbiota between the batches tested in this study had little impact on the course of the fermentation (Figure 2B). Both *L. sakei* as *P. acidilactici* were able to dominate the fermentation when larvae from a different production batch were fermented, with their relative abundances ranging between 92.2% and 98.7%. However, when sodium nitrite was added to the paste, the starters *L. lactis* (Figure A1-B, Supporting Information) and *L. sakei* (Figure A2-B, Supporting Information) were not able to completely outcompete the endogenous microbiota. Next to DNA of the added starter, DNA from an *Enterobacteriaceae* species (ASV 19, up to 16.0%,) and a *Lactobacillus* species (ASV 5, up to 25.1%) was abundantly present. 

### 3.4. Absolute Bacterial Abundances

Illumina amplicon sequencing generates relative abundances. However, since relative abundance is expressed as a proportion of the total sample, the reliability of such estimates to reflect the actual abundance of subgroups or species is insufficient. Coupling microbial abundances with microbial quantities can be more informative in describing the microbial dynamics during fermentation. Therefore, in addition to Illumina amplicon sequencing, a qPCR approach based on the 16 rRNA gene copies was conducted to estimate the total number of bacterial cells. The estimated absolute abundances (EAA), obtained by combining the quantitative detection and the relative abundances, are shown in Figure 1C and Figure 2C. 

For the control experiment, the initial number of bacterial 16S rRNA gene copies was up to 2300 copies/µL DNA extract. Higher copy numbers, up to 40,000 copies/µL DNA extract, were obtained for the inoculated samples. During the first three days of the fermentation, the 16S rRNA gene copy numbers increased in both the control and the inoculated samples. This increase in copy numbers correlated well with the increase in microbial counts. Likewise, Jung et al. [51] reported that the total 16S rRNA copy numbers of kimchi samples increased during kimchi fermentation, from an initial value of 2.6 × 10^8^ copies/mL to the highest value of 3.0 × 10^10^ copies/mL at day 25 of the fermentation. In our study, after three days of fermentation, the copy numbers of the samples either increased, remained constant or decreased. Between the samples inoculated with different starters, significant differences could be observed in copy numbers at day three and seven of the fermentation. The highest numbers, up to 1.8 × 10^6^ copies/µL DNA extract, were obtained for the paste inoculated with *L. curvatus*. However, considerable variation in the gene copy numbers was observed in some samples, as shown by large standard deviations in Figure 1C. Moreover, significantly different gene copy numbers were also found among samples originating from different fermentations inoculated with the same starter culture (Figure 2C) and among samples inoculated with the same starter culture but with and without the addition of sodium nitrite (Figure A1-C and Figure A2-C, Supporting Information). Despite those differences, all figures show the same result: The 16S rRNA copy numbers were low at the start and increased during the first stage of the fermentations. One ASV, most likely that of the added starter culture, was dominating the inoculated samples. The presence of other ASVs, which can make up a large proportion of the total sample at the start of the fermentation as shown by Illumina sequencing, is negligible as shown in Figure 1C.

An exact recalculation of the 16S rRNA copies/µL DNA extract to log cells/g mealworm paste is difficult because the copy numbers of chromosomal 16S rRNA gene operons vary per species [37,52]. The copy numbers can vary from one to as many as 15 [53]. To date, the 16S rRNA gene copy number has only been characterized in a few LAB species. For the starters investigated in this study, *L. plantarum* [54,55] contains five copies and *L. sakei* [56] possesses seven copies of the 16S rRNA gene per genome. These data allow to make an estimation of the cell numbers per gram paste. Overall, cell numbers obtained by qPCR were lower compared to the total counts obtained by plate counts. Especially at the start of the fermentations, significant differences were observed (5.0 log cells/g compared to 6.5 log cfu/g for both starters). At day three and seven, the differences were smaller: At day three 6.9 log cells/g compared to 7.7 log cfu/g and 7.7 log cells/g compared to 8.4 log cfu/g for the pastes with *L. sakei* and *L. plantarum*, respectively, and at day seven 6.7 log cells/g compared to 7.1 log cfu/g and 7.6 log cell/g compared to 8.3 log cfu/g. The differences between the qPCR counts and the microbial counts could be due to the combination of the inherent biases of all experimental steps, such as on the one hand for qPCR, the efficiency of the DNA extraction, and PCR inhibition due to the amplification efficiency and primer specificity and on the other hand for the place counts the fact that part of the microbiota is VBNC (viable but not culturable). Panicker et al. [57] have explained similar results by the presence of PCR inhibitors in shellfish homogenates. Insects are also known to contain PCR inhibitors [58,59]. However, more research is needed to investigate whether PCR inhibitors are present in the investigated mealworm paste. 

### 3.5. Consumption of Glucose and Production of Specific Amino Acids

Similar to meat products, insects contain little fermentable sugars [60]. Therefore, the addition of free sugar is necessary to fuel the fermentation process. In meat fermentations, glucose is mostly added to about 1–2% of the total weight [17]. Higher levels of sugar promote faster fermentation, which are preferred for example in the United States. In contrast, in Europe less tanginess and more diverse flavour development are generally preferred. To this end, a sugar concentration as low as 0.75% (*w*/*w*) was used in this study. With UPLC-MS, glucose was found in nearly equimolar concentrations (±1.7 g/100 g of paste) at the start of the experiments with either control or inoculated samples. Free sugars, such as glucose, play important roles in the taste of fermented food products as they are not only sweeteners but also serve as carbon sources for microorganisms, including LAB, to produce various products [51]. After seven days of fermentation, glucose was completely metabolized in all inoculated samples, whereas for the control experiments the residual glucose concentration was up to 0.1 g/100 g of paste.

As taste is an important driver of food choice, improving the sensory experience of insects is of great importance if they are to be eaten regularly. Introducing the umami taste in insects might stimulate their acceptance and consumption, as this taste is well appreciated in the meat consuming Western culture. In fermented meat products, the umami taste is mainly attributed to the presence of the free amino acids Glu and Asp [7]. The changes in the contents of those FAA observed in the mealworm pastes during fermentation are given in Table 2. On day zero, the free Asp and Glu contents of the pastes were 4.22 ± 1.00 mg/100 g of paste and 17.76 ± 3.10 mg/100 g of paste, respectively. Lower initial values (0.28 ± 0.04 mg/100 g and 7.65 ± 0.99 mg/100 g for free Asp and Glu, respectively) were reported by Limsuwan, Visessanguan, and Kongkiattikajorn [61], who investigated the effect of different starter cultures on the FAA contents in Nham, a Thai fermented pork sausage. As shown in Table 2, the use of different starter cultures resulted in different contents of the FAA Asp and Glu. More specifically, after seven days of fermentation, the free Asp content increased in the samples inoculated with the starter cultures *L. lactis*, *L. farciminis,* and *L. plantarum*, remained constant in the control experiments and the pastes inoculated with *L. curvatus*, and decreased when the starter cultures *L. sakei* and *P. acidilactici* were used. The free Glu content, on the other hand, increased when the pastes were inoculated with the starters *L. curvatus*, *L. farciminis*, *L. sakei,* or *P. acidilactici*, remained constant in the control experiments and the pastes inoculated with *L. plantarum*, and decreased when the starter *L. lactis* was added to the pastes. Similar trends were observed when larvae from a different production batch (batch 2) were fermented, as shown in Table 2. Overall, the highest total increase of the free amino acids Asp and Glu (±25 mg/100 g sample) was observed when *L. farciminis* was used. The free Glu contents (35.56 ± 1.91 mg/100 g) and the free Asp contents (10.43 ± 0.85 mg/100 g) of the pastes inoculated with this starter were substantially higher compared to the free Glu and Asp contents of non-fermented meat products, such as pork (±9 and ±3 mg/100 g, respectively) and beef (±10 and ±1 mg/100 g, respectively). Chicken, however, has generally a lower free Glu content (±22 mg/100 g) but a higher Asp content (±15 mg/100 g) [62]. Nevertheless, sensory analysis by a taste panel could now be the next step to determine whether the increase in FFA during fermentation enhances the umami flavour of the pastes. In addition, more research is needed to investigate the effect of the curing agent sodium nitrite on the content of the FAA. When sodium nitrite was added to the paste, the highest increase of the FAA Glu and Asp was observed in the pastes inoculated with *P. acidilactici*, *L. sakei,* and *L. farciminis* (Table A2, Supporting Information). 

## 4. Conclusions

In this study, the applicability of several candidate starter cultures to ferment a mealworm paste was investigated through microbiological and metabolite kinetic analyses. The results presented in this paper showed that all tested starter cultures were able to ferment pulverized mealworms. Moreover, the addition of a starter culture was required to obtain a rapid acidification and to inhibit undesirable microorganisms during fermentation. Whereas the fermentations of two of the six starters were repeated using the same batch of mealworms, the repeatability of the fermentations needs further investigation, to assess the consistency of the starters’ performance not only within the same batch of larvae but also over different batches. From all starter cultures tested, *L. farciminis* was found to be the most promising. Its application resulted in the largest increase in the content of the free amino acids Asp and Glu. This increase could be attributed to starter culture activity and might affect the organoleptic characteristics of the pastes. In a next step, it needs to be investigated whether fermentation with this starter culture may actually enhance the flavour of the mealworm paste. Driven by the consumers’ demand for more natural or organic meat products, the necessity of sodium nitrite, as often applied in traditional meat fermentation, during mealworm fermentation was questioned as well. Although in previous research nitrite proved to assist in preventing growth of unwanted background microbiota, in this study, the addition of sodium nitrite was not necessary. On the contrary, the addition of sodium nitrite delayed the growth of several starter cultures, and concomitantly reduced acidification and amino acid production. Nevertheless, more research is required to determine whether fermentation can extend the shelf life during cold storage of the fermented product and it is possible that nitrite has a positive effect on this aspect.

## Figures and Tables

**Table 1:** Microbial community diversity indices of fermented mealworm paste subjected to metagenetic analysis (without the addition of sodium nitrite).

		Observed ASV ^1^ Richness			Shannon-Wiener ^2^		
		Day 0	Day 3	Day 7	Day 0	Day 3	Day 7
Batch 1	Control	92.33 ± 10.21 ^a^	36.00 ± 4.58 ^b^	40.00 ± 12.17 ^b^	2.15 ± 0.18 ^a^	1.23 ± 0.14 ^b^	1.26 ± 0.26 ^b^
	*Lactococcus lactis*	68.33 ± 12.58 ^a^	34.00 ± 5.20 ^b^	27.00 ± 6.08 ^b^	2.07 ± 0.13 ^a^	0.33 ± 0.13 ^b^	0.17 ± 0.05 ^b^
	*Lactobacillus curvatus*	63.00 ± 9.17 ^a^	15.33 ± 4.62 ^b^	11.33 ± 0.58 ^b^	0.92 ± 0.01 ^a^	0.04 ± 0.00 ^b^	0.03 ± 0.00 ^b^
	*Lactobacillus farciminis*	78.33 ± 9.24 ^a^	19.00 ± 4.00 ^b^	21.00 ± 1.73 ^b^	1.95 ± 0.04 ^a^	0.17 ± 0.10 ^b^	0.06 ± 0.01 ^b^
	*Lactobacillus plantarum*	100.00 ± 21.70 ^a^	10.67 ± 1.53 ^b^	8.33 ± 1.53 ^b^	2.35 ± 0.34 ^a^	0.02 ± 0.00 ^b^	0.02 ± 0.00 ^b^
	*Lactobacillus sakei*	70.33 ± 3.79 ^a,A^	23.00 ± 3.61 ^b,A^	29.00 ± 6.08 ^b,A^	1.89 ± 0.02 ^a,A^	0.18 ± 0.02 ^b,A^	0.18 ± 0.05 ^b,A^
	*Pediococcus acidilactici*	74.00 ± 3.61 ^a,C^	17.67 ± 0.58 ^b,C^	16.00 ± 4.36 ^b,C^	2.15 ± 0.05 ^a,C^	0.07 ± 0.03 ^b,C^	0.07 ± 0.05 ^b,C^
Batch 2	*Lactobacillus sakei*	101.67 ± 21.22 ^a,B^	45.00 ± 2.65 ^b,B^	42.33 ± 3.51 ^b,B^	2.87 ± 0.07 ^a,B^	0.48 ± 0.05 ^b,B^	0.42 ± 0.06 ^b,B^
	*Pediococcus acidilactici*	97.33 ± 23.44 ^a,C^	36.00 ± 11.36 ^b,D^	30.00 ± 1.00 ^b,D^	2.87 ± 0.05 ^a,D^	0.28 ± 0.23 ^b,D^	0.10 ± 0.00 ^b,C^

^a,b^ Values per diversity index with the same letter do not differ significantly (*p* > 0.05) between the sampling moments of a fermentation. ^A,B^ Values per diversity index with the same letter do not differ significantly (*p* > 0.05) between different batches of *L. sakei*. ^C,D^ Values per diversity index with the same letter do not differ significantly (*p* > 0.05) between different batches of *P. acidilactici*. ^1^ ASV: Amplicon sequence variant. ^2^ Shannon-Wiener diversity index: an index to characterize species diversity based on species richness as well as their relative abundance. A higher value represents more diversity [36].

**Table 2:** Changes in the contents of the free amino acids aspartic acid (Asp) and glutamic acid (Glu) during fermentation of a mealworm paste without the addition of the curing agent sodium nitrite ^1^.

		Batch 1		Batch 2	
		Asp ^2^	Glu ^2^	Asp ^2^	Glu ^2^
Day 0		4.22 ± 1.00	17.76 ± 3.10	3.27 ± 0.62	18.64 ± 3.49
Day 7	Control	4.77 ± 0.57	20.72 ± 0.99		
	*Lactococcus lactis*	13.06 ± 0.68	0.81 ± 0.07		
	*Lactobacillus curvatus*	3.22 ± 0.06	26.52 ± 0.20		
	*Lactobacillus farciminis*	10.43 ± 0.85	35.56 ± 1.91		
	*Lactobacillus plantarum*	7.85 ± 0.42	19.07 ± 0.13		
	*Lactobacillus sakei*	0.73 ± 0.20	24.92 ± 5.25	1.76 ± 0.01	33.08 ± 0.22
	*Pediococcus acidilactici*	1.31 ± 0.02	37.09 ± 1.14	2.37 ± 0.11	34.95 ± 0.35

^1^ Values are the mean of two replicate samples ± standard deviation. ^2^ Results are expressed as mg/100 g of paste.

## Appendix A

**Table A1:** **(Supporting Information).** Microbial community diversity indices of fermented mealworm paste subjected to metagenetic analysis (with the addition of sodium nitrite).

		Observed ASV ^1^ Richness			Shannon-Wiener ^2^		
		Day 0	Day 3	Day 7	Day 0	Day 3	Day 7
Batch 1	Control	78.67 ± 2.52 ^a^	30.33 ± 2.31 ^b^	28.00 ± 4.00 ^b^	2.02 ± 0.17 ^a^	0.89 ± 0.05 ^b^	0.77 ± 0.19 ^b^
	*Lactococcus lactis*	77.33 ± 7.09 ^a^	43.67 ± 8.08 ^b^	43.33 ± 23.09 ^b^	2.03 ± 0.11 ^a^	0.96 ± 0.17 ^b^	0.95 ± 0.21 ^b^
	*Lactobacillus curvatus*	47.00 ± 2.83 ^a^	22.33 ± 2.08 ^b^	20.00 ± 3.46 ^b^	0.89 ± 0.04 ^a^	0.17 ± 0.01 ^b^	0.11 ± 0.02 ^b^
	*Lactobacillus farciminis*	83.00 ± 10.00 ^a^	22.33 ± 5.03 ^b^	16.67 ± 4.04 ^b^	1.91 ± 0.05 ^a^	0.07 ± 0.02 ^b^	0.04 ± 0.01 ^b^
	*Lactobacillus plantarum*	87.33 ± 9.02 ^a^	30.67 ± 3.06 ^b^	20.33 ± 2.08 ^b^	2.18 ± 0.09 ^a^	0.14 ± 0.01 ^b^	0.06 ± 0.01 ^b^
	*Lactobacillus sakei*	73.33 ± 19.60 ^a,A^	24.00 ± 4.58 ^b,A^	20.33 ± 2.08 ^b,A^	1.87 ± 0.07 ^a,A^	0.16 ± 0.02 ^b,A^	0.13 ± 0.01 ^b,A^
	*Pediococcus acidilactici*	71.33 ± 3.21 ^a,C^	43.67 ± 5.51 ^b,C^	33.33 ± 4.51 ^b,C^	1.83 ± 0.05 ^a,C^	0.31 ± 0.07 ^b,C^	0.19 ± 0.03 ^b,C^
Batch 2	*Lactobacillus sakei*	116.00 ± 12.77 ^a,B^	51.67 ± 8.33 ^b,B^	56.67 ± 10.60 ^b,B^	2.83 ± 0.06 ^a,B^	0.88 ± 0. 41 ^b,B^	1.16 ± 0.32 ^b,B^
	*Pediococcus acidilactici*	89.00 ± 17.35 ^a,D^	42.67 ± 11.37 ^b,C^	39.67 ± 0.58 ^b,D^	2.82 ± 0.05 ^a,D^	0.46 ± 0.29 ^b,D^	0.40 ± 0.08 ^b,D^

^a,b^ Values per diversity index with the same letter do not differ significantly (*p* > 0.05) between the sampling moments of a fermentation. ^A,B^ Values per diversity index with the same letter do not differ significantly (*p* > 0.05) between different batches of *L. sakei*. ^C,D^ Values per diversity index with the same letter do not differ significantly (*p* > 0.05) between different batches of *P. acidilactici*. ^1^ ASV: Amplicon sequence variant. ^2^ Shannon-Wiener diversity index: an index to characterize species diversity based on species richness as well as their relative abundance. A higher value represents more diversity [36].

**Table A2:** **(Supporting Information)**. Changes in the contents of the free amino acids aspartic acid (Asp) and glutamic acid (Glu) during fermentation of a mealworm paste with the addition of the curing agent sodium nitrite^1^.

		Batch 1		Batch 2	
		Asp ^2^	Glu ^2^	Asp ^2^	Glu ^2^
Day 0		4.32 ± 0.89	18.06 ± 2.97	4.05 ± 0.46	21.08 ± 3.55
Day 7	Control	2.64 ± 0.23	16.43 ± 1.49		
	*Lactococcus lactis*	14.83 ± 1.77	21.08 ± 0.90		
	*Lactobacillus curvatus*	1.85 ± 0.20	26.11 ± 3.65		
	*Lactobacillus farciminis*	9.72 ± 0.42	29.31 ± 0.82		
	*Lactobacillus plantarum*	4.84 ± 0.42	19.04 ± 0.36		
	*Lactobacillus sakei*	1.23 ± 0.09	32.66 ± 1.83	2.68 ± 0.32	36.70 ± 2.76
	*Pediococcus acidilactici*	1.80 ± 0.14	37.12 ± 0.09	2.91 ± 0.27	32.80 ± 0.81

^1^ Values are the mean of two replicate samples ± standard deviation. ^2^ Results are expressed as mg/100 g of paste.

## References

[1] De Smet J., Lenaerts S., Borremans A., Scholliers J., Van Der Borght M., Van Campenhout L. (2019). Stability assessment and laboratory scale fermentation of pastes produced on a pilot scale from mealworms (*Tenebrio molitor*). LWT.

[2] Deroy O., Reade B., Spence C. (2015). The insectivore’s dilemma, and how to take the West out of it. Food Qual. Prefer..

[3] Hartmann C., Shi J., Giusto A., Siegrist M. (2015). The psychology of eating insects: A cross-cultural comparison between Germany and China. Food Qual. Prefer..

[4] Schouteten J.J., De Steur H., De Pelsmaeker S., Lagast S., Juvinal J.G., De Bourdeaudhuij I., Verbeke W., Gellynck X. (2016). Emotional and sensory profiling of insect-, plant-, and meat-based burgers under blind, expected and informed conditions. Food Qual. Prefer..

[5] Tan H.S.G., Fischer A.R.H., Tinchan P., Stieger M., Steenbekkers L.P.A., van Trijp H.C.M. (2015). Insects as food: Exploring cultural exposure and individual experience as determinants of acceptance. Food Qual. Prefer..

[6] Laranjo M., Potes M.E., Elias M. (2019). Role of starter cultures on the safety of fermented meat products. Front. Microbiol..

[7] Zhao C.J., Schieber A., Gänzle M.G. (2016). Formation of taste-active amino acids, amino acid derivatives and peptides in food fermentation—A review. Food Res. Int..

[8] Klunder H.C., Wolkers-Rooijackers J., Korpela J.M., Nout M.J.R. (2012). Microbiological aspects of processing and storage of edible insects. Food Control.

[9] Borremans A., Lenaerts S., Crauwels S., Lievens B., Van Campenhout L. (2018). Marination and fermentation of yellow mealworm larvae (*Tenebrio molitor*). Food Control.

[10] Hugas M., Monfort J.M. (1997). Bacterial starter cultures for meat fermentation. Food Chem..

[11] Leroy F., De Vuyst L. (2004). Lactic acid bacteria as functional starter cultures for the food fermentation industry. Trends Food Sci. Technol..

[12] Fadda S., López C., Vignolo G. (2010). Role of lactic acid bacteria during meat conditioning and fermentation: Peptides generated as sensorial and hygienic biomarkers. Meat Sci..

[13] Katla A.K., Kruse H., Johnsen G., Herikstad H. (2001). Antimicrobial susceptibility of starter culture bacteria used in Norwegian dairy products. Int. J. Food Microbiol..

[14] Castellano P., Belfiore C., Fadda S., Vignolo G. (2008). A review of bacteriocinogenic lactic acid bacteria used as bioprotective cultures in fresh meat produced in Argentina. Meat Sci..

[15] Verluyten J., Messens W., de Vuyst L. (2003). The curing agent sodium nitrite, used in the production of fermented sausages, is less inhibiting to the bacteriocin-producing meat starter culture Lactobacillus curvatus LTH 1174 under anaerobic conditions. Appl. Environ. Microbiol..

[16] Nowak V., Persijn D., Rittenschober D., Charrondiere U.R. (2016). Review of food composition data for edible insects. Food Chem..

[17] Hutkins R.W. (2006). Microbiology and Technology of Fermented Foods.

[18] Dijk R., van den Berg D., Beumer R., de Boer E., Dijkstra A., Mout L., Stegeman H., Uyttendaele M., In’t Veld S. (2015). Microbiologie van Voedingsmiddelen: Methoden, Principes en Criteria.

[19] Kozich J.J., Westcott S.L., Baxter N.T., Highlander S.K., Schloss P.D. (2013). Development of a dual-index sequencing strategy and curation pipeline for analysing amplicon sequence data on the MiSeq Illumina Sequencing platform. Appl. Environ. Microbiol..

[20] Caporaso J.G., Lauber C.L., Walters W.A., Berg-Lyons D., Lozupone C.A., Turnbaugh P.J., Fierer N., Knight R. (2011). Global patterns of 16S rRNA diversity at a depth of millions of sequences per sample. Proc. Natl. Acad. Sci. USA.

[21] Edgar R.C. (2013). UPARSE: Highly accurate OTU sequences from microbial amplicon reads. Nat. Methods.

[22] Schloss P.D., Westcott S.L., Ryabin T., Hall J.R., Hartmann M., Hollister E.B., Lesniewski R.A., Oakley B.B., Parks D.H., Robinson C.J. (2009). Introducing Mothur: Open-source, platform-independent, community-supported software for describing and comparing microbial communities. Appl. Environ. Microbiol..

[23] Gurevich A., Saveliev V., Vyahhi N., Tesler G. (2013). Genome analysis QUAST: Quality assessment tool for genome assemblies. Bioinformatics.

[24] Edgar R.C. (2016). UNOISE2: Improved error-correction for Illumina 16S and ITS 581 amplicon sequencing. bioRxiv.

[25] Edgar R.C. (2016). SINTAX: A simple non-Bayesian taxonomy classifier for 16S and ITS583 sequences. bioRxiv.

[26] Altschul S., Gish G., Miller W., Myers E.W., Lipman D. (1990). Basic local alignment search tool. J. Mol. Biol..

[27] Benson D.A., Karsch-Mizrachi I., Lipman D.J., Ostell J., Wheeler D.L. (2013). GenBank. Nucleic Acids Res..

[28] Leroy F., Verluyten J., De Vuyst L. (2006). Functional meat starter cultures for improved sausage fermentation. Int. J. Food Microbiol..

[29] Vandeweyer D., Crauwels S., Lievens B., Van Campenhout L. (2017). Microbial counts of mealworms (*Tenebrio molitor*) and crickets (*Acheta domesticus* and *Gryllodes sigillatus*) from different rearing companies and different production batches. Int. J. Food Microbiol..

[30] Korkeala H., Alanko T., Tiusanen T. (1992). Effect of sodium nitrite and sodium chloride on growth of lactic acid bacteria. Acta Vet. Scand..

[31] Verluyten J., Leroy F., de Vuyst L. (2004). Effects of different spices used in production of fermented sausages on growth of and curvacin A production *of Lactobacillus curvatus* LTH 1174. Appl. Environ. Microbiol..

[32] Vandeweyer D., Lenaerts S., Callens A., Van Campenhout L. (2017). Effect of blanching followed by refrigerated storage or industrial microwave drying on the microbial load of yellow mealworms (*Tenebrio molitor*). Food Control.

[33] Leroy F., De Vuyst L. (1999). Temperature and pH conditions that prevail during fermentation of sausages are optimal for production of the antilisterial bacteriocin sakacin K. Appl. Environ. Microbiol..

[34] Leroy F., Lievens K., de Vuyst L. (2005). Interactions of meat-associated bacteriocin-producing Lactobacilli with *Listeria* innocula under stringent sausage fermentation conditions. J. Food Protect..

[35] Sriphochanart W., Skolpap W. (2010). The use of selected lactic acid bacteria starter cultures for improved Thai sausage fermentation. J. Food Process. Preserv..

[36] Shannon C. (1948). A mathematical theory of communication. Bell Syst. Tech. J..

[37] Farrelly V., Rainley F.A., Stackebrandt E. (1995). Effect of genome size and rrn gene copy number in PCR amplification of 16S rRNA genes from a mixture of bacterial species. Appl. Environ. Microbiol..

[38] Garofalo C., Osimani A., Milanovic V., Taccari M., Cardinali F., Aquilanti L., Riolo P., Ruschioni S., Isidoro N., Clementi F. (2017). The microbiota of marketed processed edible insects as revealed by high-throughput sequencing. Food Microbiol..

[39] Jung J., Heo A., Park Y.W., Kim Y.J., Koh H., Park W. (2014). Gut microbiota of *Tenebrio molitor* and their response to environmental change. J. Microbiol. Biotechnol..

[40] Wang Y., Zhang Y. (2015). Investigation of gut-associated bacteria in *Tenebrio molitor* (Coleoptera: Tenebrionidae) larvae using culture-dependent and DGGE methods. Ann. Entomol. Soc. Am..

[41] Vandeweyer D., Crauwels S., Lievens B., Van Campenhout L. (2017). Metagenetic analysis of the bacterial communities of edible insects from diverse production cycles at industrial rearing companies. Int. J. Food Microbiol..

[42] Madigan M.T., Martinko J.M., Dunlap P.V., Clark D.P. (2009). Brock Biology of Microorganisms.

[43] Baylis C., Uyttendaele M., Joosten H., Davies A. (2011). The Enterobacteriaceae and Their Significance to the Food Industry.

[44] Osimani A., Milanović V., Cardinali F., Garofalo C., Clementi F., Pasquini M., Riolo P., Ruschioni S., Isidoro N., Loreto N. (2018). The bacterial biota of laboratory-reared edible mealworms (*Tenebrio molitor* L.): From feed to frass. Int. J. Food Microbiol..

[45] Stoops J., Crauwels S., Waud M., Claes J., Lievens B., Van Campenhout L. (2016). Microbial community assessment of mealworms (*Tenebrio molitor*) and grasshoppers (*Locusta migratoria migratorioides*) sold for human consumption. Food Microbiol..

[46] M’hir S., Minervini F., Di Cagno R., Chammem N., Hamdi M. (2012). Technological, functional and safety aspects of enterococci in fermented vegetable products: A mini-review. Ann. Microbiol..

[47] Pot B., Felis G.E., De Bruyne K., Tsakalidou E., Papadimitriou K., Leisner J., Vandamme P., Holzapfel W.P., Wood B.J.B. (2014). The genus *Lactobacillus*. Lactic Acid Bacteria: Biodiversity and Taxonomy.

[48] Zheng J., Ruan L., Sun M., Gänzle M. (2015). A genomic view of *lactobacilli* and *pediococci* demonstrates that phylogeny matches ecology and physiology. Appl. Environ. Microbiol..

[49] Sun Z., Harris H.M., McCann A., Guo C., Argimón S., Zhang W., Yang X., Jeffery I.B., Cooney J.C., Kagawa T.F. (2015). Expanding the biotechnology potential of *lactobacilli* through comparative genomics of 213 strains and associated genera. Nat. Commun..

[50] Wang L.T., Lee F.L., Tai C.J., Kasai H. (2007). Comparison of gyrB gene sequences, 16S rRNA gene sequences and DNA–DNA hybridization in the *Bacillus subtilis* group. Int. J. Syst. Evol. Microbiol..

[51] Jung J.Y., Lee S.H., Kim J.M., Park M.S., Bae J.W., Hahn Y., Madsen E.L., Jeon C.O. (2011). Metagenomic analysis of kimchi, a traditional Korean fermented food. Appl. Environ. Microbiol..

[52] Park E.J., Chang H.W., Kim K.H., Nam Y.D., Roh S.W., Bae J.W. (2009). Application of quantitative real-time PCR for enumeration of total bacterial, archaeal, and yeast populations in kimchi. J. Microbiol..

[53] Acinas S.G., Marcelino L.A., Klepac-Ceraj V., Polz M.F. (2004). Divergence and redundancy of 16S rRNA sequences in genomes with multiple rrn operons. J. Bacteriol..

[54] Chevallier B., Hubert J.C., Kammerer B. (1994). Determination of chromosome size and number of rrn loci in *Lactobacillus plantarum* by pulse-field gel electrophoresis. FEMS Microbiol. Lett..

[55] Kleerebezem M., Boekhorst J., van Kranenburg R., Molenaar D., Kuipers O.P., Leer R., Tarchini R., Peters S.A., Sandbrink H.M., Fiers M.W. (2003). Complete genome sequence of *Lactobacillus plantarum* WCFS1. Proc. Natl. Acad. Sci. USA.

[56] Dudez A.M., Chaillou S., Hissler L., Stentz R., Champomier-Vergès M.C., Alpert C.A., Zagorec M. (2002). Physical and genetic map of the *Lactobacillus sakei* 23K chromosome. Microbiology.

[57] Panicker G., Myers M.L., Bej A.K. (2004). Rapid detection of *Vibrio vulnificus* in shellfish and gulf water by real-time PCR. Appl. Environ. Microbiol..

[58] Boncristiani H., Li J., Evans J.D., Pettis J., Chen Y. (2011). Scientific note on PCR inhibitors in the compound eyes of honey bees, *Apis Mellifera*. Apidologie.

[59] Shamim G., Ranjan S.K., Pandey D.M., Ramani R. (2014). Biochemistry and biosynthesis of insect pigments. Eur. J. Entomol..

[60] Morales-Ramos J.A., Guadalupe Rojas M., Shelby K., Coudron T.A. (2016). Nutritional value of pupae versus larvae of *Tenebrio molitor* (Coleoptera: Tenebrionidae) as food for rearing *Podisus maculiventris* (Heteroptera: Pentatomidae). J. Econ. Entomol..

[61] Limsuwan S., Visessanguan W., Kongkiattikajorn J. (2007). The effects of starter cultures on biogenic amine and free amino acid contents in Nham during fermentation. Nat. Sci..

[62] Triki M., Herrero A.M., Jiménez-Colmenero F., Ruiz-Capillas C. (2018). Quality assessment of fresh meat from several species based on free amino acid and biogenic amine contents during chilled storage. Foods.

